# Quantitative Hybrid Cardiac [^18^F]FDG-PET-MRI Images for Assessment of Cardiac Repair by Preconditioned Cardiosphere-Derived Cells

**DOI:** 10.1016/j.omtm.2020.06.008

**Published:** 2020-06-15

**Authors:** Johannes Winkler, Dominika Lukovic, Julia Mester-Tonczar, Katrin Zlabinger, Alfred Gugerell, Noemi Pavo, András Jakab, Zsuzsanna Szankai, Denise Traxler, Claudia Müller, Andreas Spannbauer, Martin Riesenhuber, Ena Hašimbegović, James Dawkins, Matthias Zimmermann, Hendrik J. Ankersmit, Eduardo Marbán, Mariann Gyöngyösi

**Affiliations:** 1 Department of Cardiology, Medical University of Vienna, Vienna, Austria; 2 Department of Biomedical Imaging and Image-guided Therapy, Medical University of Vienna, Währinger Gürtel 18-20, 1090 Vienna, Austria; 3 Center for MR-Research, University Children’s Hospital Zurich, Steinwiesstrasse 7e, 80cb Zurich, Switzerland; 4 Smidt Heart Institute, Cedars-Sinai Medical Center, Los Angeles, CA, USA; 5 Division of Thoracic Surgery, Medical University of Vienna, Vienna, Austria

**Keywords:** heart failure, magnetic resonance imaging, myocardial regeneration, nuclear cardiology, PET, stem cells, large animal model, proteomics

## Abstract

Cardiosphere-derived cells (CDCs) are progenitor cells derived from heart tissue and have shown promising results in preclinical models. APOSEC, the secretome of irradiated peripheral blood mononuclear cells, has decreased infarct size in acute and chronic experimental myocardial infarction (MI). We enhanced the effect of CDCs with APOSEC preconditioning (apoCDC) and investigated the reparative effect in a translational pig model of reperfused MI. Supernatants of CDCs, assessed by proteomic analysis, revealed reduced production of extracellular matrix proteins after *in vitro* APOSEC preconditioning. In a porcine model of catheter-based reperfused anterior acute MI (AMI), CDCs with (apoCDC, n = 8) or without APOSEC preconditioning (CDC, n = 6) were infused intracoronary, 15 min after the start of reperfusion. Untreated AMI animals (n = 7) and sham procedures (n = 5) functioned as controls. 2-deoxy-2-(18 F)-fluoro-D-glucose-positron emission tomography-magnetic resonance imaging ([^18^F]FDG-PET-MRI), with late enhancement after 1 month, showed reduced scar volume and lower transmurality of the infarcted area in CDC and apoCDC compared to AMI controls. Segmental quantitative PET images displayed indicated more residual viability in apoCDC. The left-ventricle (LV) ejection fraction was improved nonsignificantly to 45.8% ± 8.6% for apoCDC and 43.5% ± 7.1% for CDCs compared to 38.5% ± 4.4% for untreated AMI. Quantitative hybrid [^18^F]FDG-PET-MRI demonstrated improved metabolic and functional recovery after CDC administration, whereas apoCDCs induced preservation of viability of the infarcted area.

## Graphical Abstract

**Figure:**
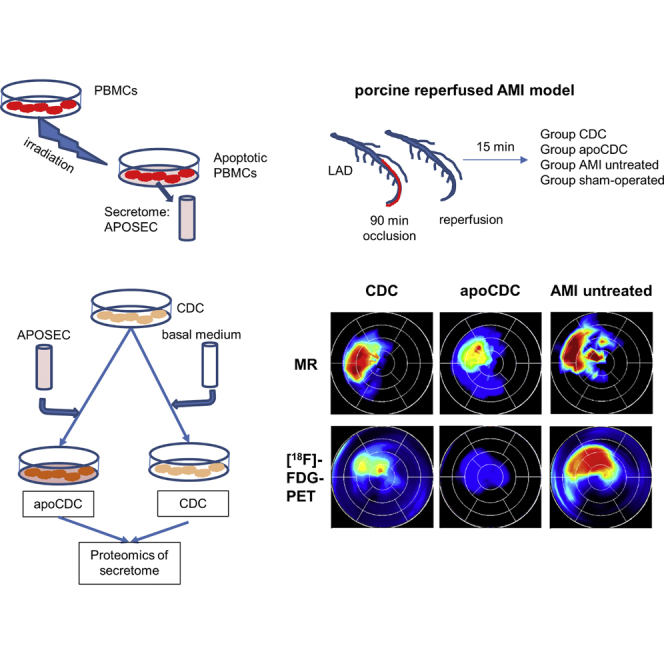

## Teaser

Paracrine effects are regarded to be decisive for the success of stem cells for cardiac repair. Winkler and colleagues show that *in vitro* conditioning with the cell-protective and prosurvival mixture APOSEC alters the paracrine signature of cardiosphere-derived cells (CDCs). In a translational porcine AMI model, APOSEC-conditioned CDCs improved myocardial viability and decreased transmurality in the infarct area, as assessed by hybrid PET-MRI.

## Introduction

After almost 20 years of intense research and recent setbacks in clinical evaluations, the utility, risks, and efficacy of intracoronary cell administration are controversially debated.^1^^,^^2^ The main, generally accepted mechanism of eventual improvement in cardiac function in experimental and some clinical studies is a paracrine effect caused by secreted factors.^3^ A recent report identified a contribution of an acute immune response through induction of macrophages to the functional recovery after myocardial infarction (MI).^4^ Cardiac progenitor cells appear to have superior effects compared to mesenchymal stem cells, the latter of which are easily available and frequently used in clinical trials^5^ but have overall yielded insufficient clinical benefit.

Cardiosphere-derived cells (CDCs) are a mixture of mesenchymal, stromal, and progenitor cells derived from cultures of myocardial biopsies.^6^ Preclinical and clinical studies demonstrated that intracoronary delivery of allogeneic CDCs, directly after reperfusion of acute MI (AMI), is effective in cardioprotection, prevents microvascular obstruction, and attenuates adverse acute remodeling.^7^^,^^8^

It has been demonstrated that CDCs induce cardiac reparative mechanisms, despite only minimal direct cardiomyogenic differentiation, which fails to contribute meaningfully to their beneficial effects.^9^ Their therapeutic activity is mainly due to distinctive impacts on macrophages and paracrine effects, mediated by a mixture of cytokines, including angiogenic and homing factors,^5^ but also by exosomal contents, such as microRNAs (miRNAs).^10^ The cardiac repair capacity of CDCs was assessed in randomized clinical trials in humans (Cardiosphere-Derived Autologous Stem Cells to Reverse Ventricular Dysfunction [CADUCEUS] and Intracoronary Allogeneic Heart Stem Cells to Achieve Myocardial Regeneration [ALLSTAR]) in the subacute phase of AMI in a limited number of patients with initially promising results in terms of a decrease in infarct size, which, however, did not result in a significant increase in global left-ventricle (LV) ejection fraction (EF).^11^ Although the efficacy of CDCs after infarction appears not to be sufficiently robust for clinical application, the modulation and enhancement of secreted factors of CDCs are still attractive strategies for improving cell-based therapeutic implications.

Previous studies have demonstrated that administration of the secretome of apoptotic peripheral blood cells (cell-free reparative therapy; APOSEC; containing soluble factors derived from γ-irradiated peripheral blood mononuclear cells [PBMCs]) results in cytoprotection in the acute and chronic phase of AMI.^12^^,^^13^ In a large animal AMI model, APOSEC prevented ventricular remodeling, abrogated platelet aggregation, and promoted vasodilatation.^14^ The effect of APOSEC was shown to be caused by a combination of different factors, including proteins, vesicle-derived short noncoding RNAs, and lipids. Separate fractions, as well as secretomes of PBMC subpopulations, failed to replicate completely the effects regarding tissue repair, wound healing, and angiogenesis.^15^, ^16^, ^17^

Accordingly, we hypothesized that (1) preconditioning of CDCs with APOSEC (apoCDC) enhances the survival and reparative function of CDCs by influencing the paracrine effects through relevant cardioregenerative factors and cytokines and (2) cardiac magnetic resonance imaging (MRI), for assessing the beneficial effect in cardiac regeneration studies, is insufficiently sensitive for displaying increased myocardial cell viability and perfusion. Therefore, we injected apoCDCs to investigate a cardioprotective effect of intracoronary delivery of allogeneic apoCDCs directly after reperfusion of AMI and compared the LV function and myocardial viability by quantitative hybrid 2-deoxy-2-(18 F)-fluoro-D-glucose-positron emission tomography-MRI ([^18^F]FDG)-PET-MRI) images with the animals receiving nonconditioned CDCs, nontreated AMI, and sham-procedure controls.

## Results

### Coculture of APOSEC with CDCs Prevents Secretion of Extracellular Matrix (ECM) Proteins and Increases Expression and Secretion of Angiogenic and Homing Factors

**Figure 1:**
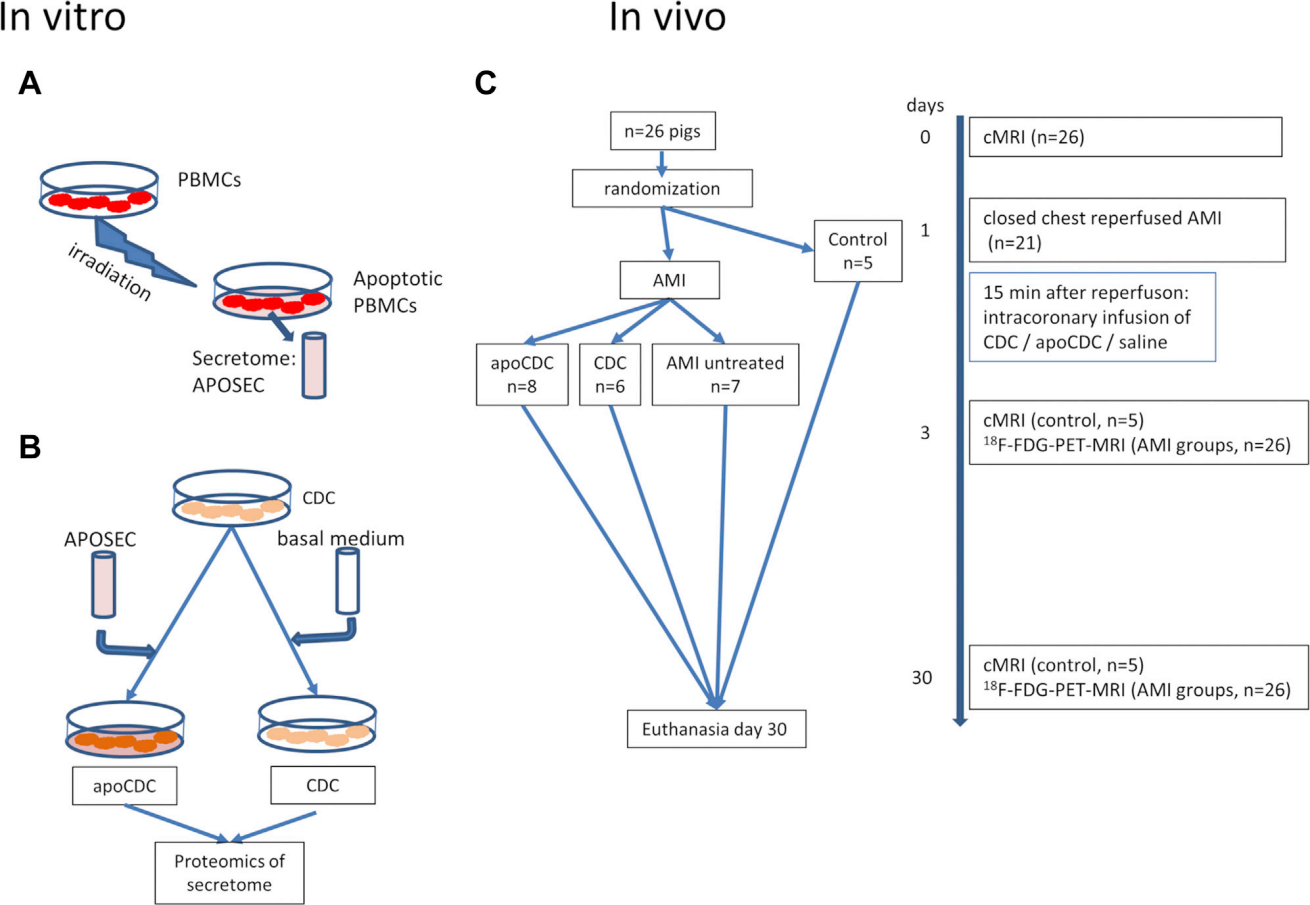
Study Design (A) Porcine PBMCs were isolated and irradiated. Apoptotic PBMCs were further incubated *in vitro* for 24 h, and the supernatant of the apoptotic PBMCs (APOSEC) was collected. (B) Porcine CDCs were incubated with (apoCDC) or without APOSEC, and the cells were stored in liquid nitrogen. The secretome of CDCs and apoCDCs was analyzed by proteomics. (C) Pigs underwent 90 min occlusion of the mid-LAD and were randomized to receive CDCs (107 cells) with or without APOSEC *in vitro* preconditioning by intracoronary infusion during the reperfusion phase. [^18^F]FDG-PET-MRI imaging was performed 3 and 30 days later, and follow-up examinations were done at indicated time points.

We investigated apoCDCs for maximizing the prosurvival and secretion-enhancing effects (Figure 1). Therefore, in a first step, we assessed the effect of APOSEC on CDC *in vitro* and examined the content of secretory proteins with or without APOSEC supplementation with a proteomic analysis. In order to facilitate the proteome analysis, cells were incubated in the absence of serum.

**Figure 2:**
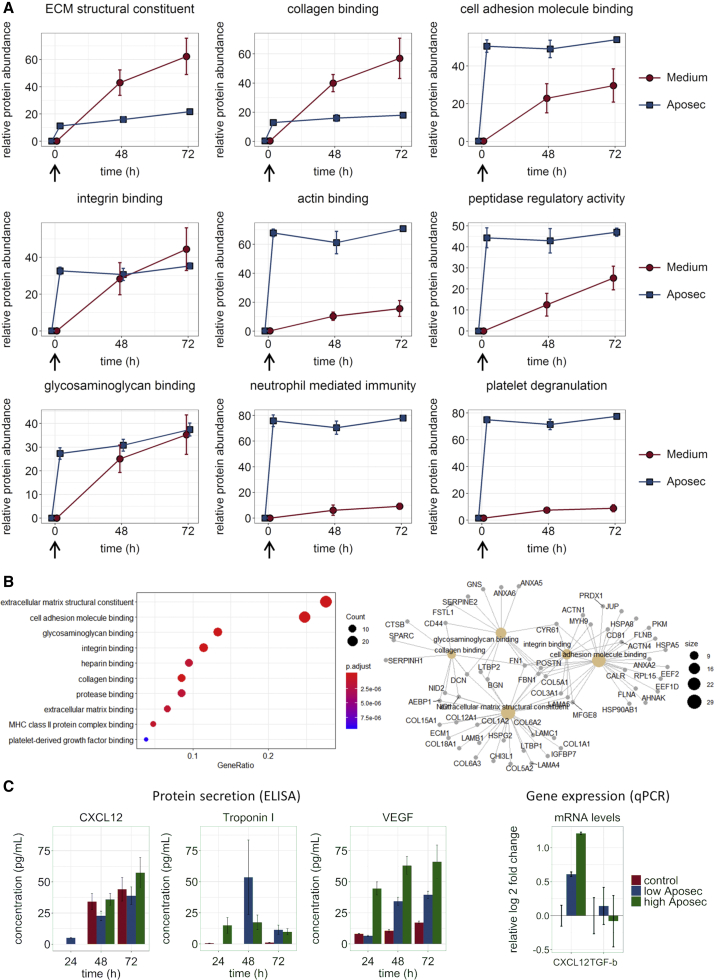
Quantitative Changes of Secreted Proteins and Effect on Gene Expression *In Vitro* in Serum-Free or APOSEC-Containing Medium CDCs were incubated with medium, with or without APOSEC, and supernatants were collected at 48 and 72 h and compared to baseline. Data are from three technical replicates for each of three independent experiments. (A) Relative individual protein quantities were assessed by ESI-MS proteomics analysis, normalized, and summarized for the indicated GO terms. Error bars are SEM. Arrows show addition of APOSEC, delineating the components of APOSEC from secreted proteins. Individual protein values with statistical evaluations are shown in Table S1. (B) GO enrichment analysis of proteins detected in the proteome analysis with significantly increased secretion by CDCs in standard medium, as opposed to APOSEC conditioning. (C) Concentration of VEGF and CXCL12 in cell-culture supernatants, measured by ELISA, and gene expression of CDCs after 72 h incubation, with or without APOSEC. Data are mean ± SD.

To distinguish between components of APOSEC and proteins secreted into the supernatant by CDCs, we analyzed quantitative protein differences between baseline and end of the incubation period. The proteomics data identified protein clusters that were secreted significantly stronger into the extracellular space by CDCs when cultured without APOSEC on the one hand and original components of APOSEC on the other hand. A continuous increase of proteins allocated to a distinct cluster during the 72-h incubation time indicates secretion by CDCs, whereas a sharp increase after addition of APOSEC signifies that the respective proteins are inherent constituents of the PBMC secretome (Figure 2; Tables 1 and S1). Regarding the reduced secretion of proteins upon incubation with APOSEC, Gene Ontology (GO) analysis showed a strong association of these proteins with ECM and cell adhesion and binding (Figure 2; Tables S2 and S3). The GO terms are overlapping, indicating a shift of the paracrine phenotype of CDCs under standard culture conditions toward secretion of ECM remodeling proteins. Several collagens, including the major myocardial collagens I and III, other ECM proteins (lamins, laminins, fibronectin, lysyl oxidase 2), and proteins that are essential for cell adhesion and protein binding, such as actinin, fibrinogen, and periostin, were secreted significantly stronger in the native cell culture (i.e., in the absence of APOSEC).

**Table 1:** Overview of *In Vitro* Secretion of Protein Clusters by CDCs in the Presence or Absence of APOSEC

GO Term	Regulated		Selected Regulated Proteins	Myocardial Role
	without APOSEC	with APOSEC		
ECM structural constituent	↑↑	→ (low)	collagen 1,3, fibronectin, fibrinogen	cardiac remodeling
Collagen binding	↑	→ (low)	decorin, nidogen, cathepsin B	cardiac remodeling
Cell adhesion molecule binding	↑	→ (high)	calreticulin, HSP70, profilin	cell-cell and cell-ECM interactions
Integrin binding	↑	→ (medium)	TIMP2, laminin alpha	ECM adhesion
Actin binding	→ (low)	→ (high)	actinin, actin-related proteins, gelsolin, profilin	cytoskeleton components
Peptidase activity	↑	→ (high)	serpins, complement C3	hemostase, fibrinolysis
Glycosaminoglycan binding	↑	↑	biglycan, apoE, periostin	ECM interactions, lipoproteins
Neutrophil-mediated immunity	→ (low)	→ (high)	aldolase, calpain, hemoglobin	immune response
Platelet degranulation	→ (low)	→ (high)	pro-platelet basic protein, alpha globulin	inflammatory processes

Time-dependent changes are presented in Figure 2. GO, gene ontology; ECM, extracellular matrix; HSP70, heat shock protein 70; TIMP2, tissue inhibitor of metalloproteinase 2.

A limited number of proteins—a few guanosine triphosphate (GTP)-binding proteins—were found to be secreted stronger by CDCs upon incubation with APOSEC (Table 1). This apparently indicates a transition of CDCs under serum-free cell-culture conditions toward a myofibroblast-like phenotype and a protecting effect of APOSEC on CDCs. An influence of serum deprivation during the incubation time might be possible. However, no detrimental effects on cell viability and morphology were detected (Figure S1).

As previously documented for human APOSEC,^16^ a considerable number of proteins were detected in porcine APOSEC-conditioned medium before the start of CDC preconditioning. The concentrations of the majority of these proteins remained constant during the 72-h incubation period; i.e., they were not further secreted by CDCs. APOSEC is produced from apoptotic PBMCs, and the expected enrichment of proteins found in neutrophils and other blood cells and proteins induced by cellular stress was confirmed (Figures S2 and S3). Together with lipids, miRNAs, extracellular vesicles (EVs), and potentially other components, these proteins contribute to the reparative capacity of APOSEC.

The electrospray ionization-mass spectrometry (ESI-MS) analysis includes proteins above the detection limit of the method. Therefore, we specifically analyzed expression and secretion of low-abundant proteins for angiogenesis (vascular endothelial growth factor [VEGF]) and stem-cell signaling (transforming growth factor β1 [TGFβ1]) and homing (C-X-C motif chemokine ligand 12 [CXCL12]). VEGF secretion into the cell-culture supernatant was increased by APOSEC conditioning in a time- and concentration-dependent manner (Figure 2). Gene expression of *CXCL12* was increased around 2-fold over CDCs cultured in standard cell-culture medium (72 h). CXCL12 protein levels in the cell-culture supernatant showed a nonsignificant increase, indicating that upregulation of CXCL12 production is slower than VEGF regulation. Taken together, these results confirmed a prosurvival and proangiogenic effect of APOSEC on CDCs and served as the basis for selecting the dose and pretreatment duration for *in vivo* evaluations.

### Effects of CDCs with and without APOSEC Preconditioning in a Large Animal AMI Model

**Figure 3:**
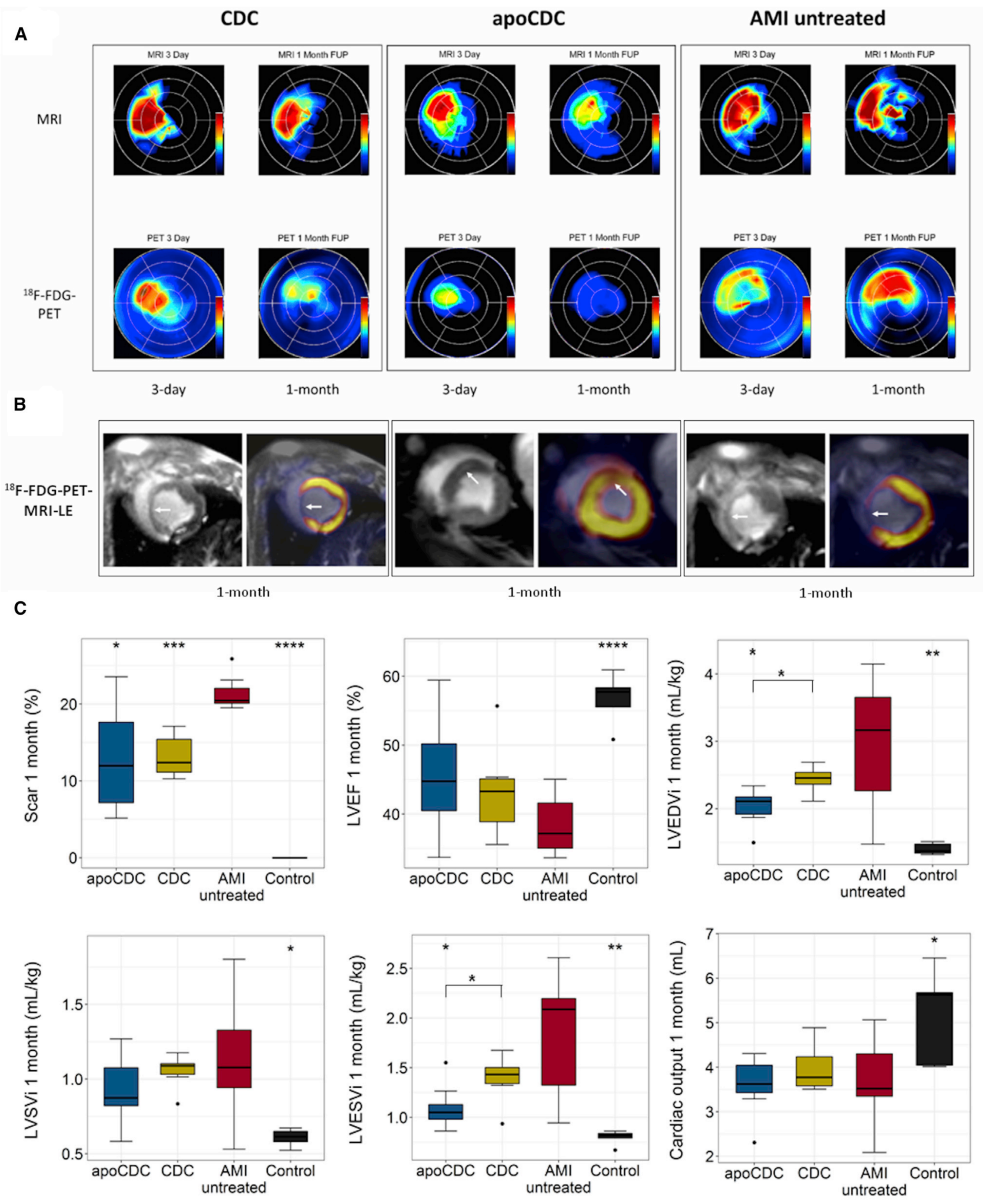
Representative [^18^F]FDG-PET-MRI Polar Map Imaging with Late Enhancement of Porcine Hearts after AMI and Treatment with apoCDCs, CDCs, or Saline (AMI Untreated) at 3 Days and 1 Month and Summary of Functional Parameters Detected by PET-MRI Imaging (A) At 3 days, similar-sized transmurality (red color) was detected in all 3 groups, with decreasing transmurality in CDC and apoCDC groups at the 1-month follow-up period (FUP). Reduced viability was found in the anteroseptal region with visual smaller size of nonviable and reduced viable areas both at 3 days and 1 month FUP in the apoCDC group. (B) MRI and MRI-PET fusion images. Group CDC (left): MRI showed myocardial thinning and increased signal intensity in the mid-anteroseptal/apical anterior segments. MRI-PET fusion image indicated reduced tracer uptake in the same region (arrows). Group apoCDC (middle): MRI showed high late-enhancement signal intensity in the apical septal segment, and MRI-PET fusion image demonstrated tracer uptake in the same region (arrows). Group AMI untreated (right): a large infarct resulted in myocardial thinning and delayed enhancement in the mid-anteroseptal and apical segments (arrows). MRI and PET image fusion was done for visualization purposes in 3D Slicer software. (C) Scar area, LV EF, end-diastolic (LV EDV index [LVEDVi]), stroke volume (LV SV index [LVSVi]), and end-systolic (LV ESV index [LVESVi]) indices relative to body weight and cardiac output were assessed by cardiac MRI, 1 month after infarction and cell treatment. ∗p < 0.05, ∗∗∗p < 0.001, ∗∗∗∗p < 0.0001 (ANOVA with Bonferroni post hoc) compared to AMI untreated (scar, LVESV, LVEDV) or between apoCDC and CDC where indicated.

We used a translational large animal model to assess effects of apoCDCs for treating AMI-induced heart failure, analyzed by cardiac [^18^F]FDG-PET-MRI with late enhancement (LE) after 3 days and 1 month. Comparative representative PET polar map images with simultaneous MRI infarct transmurality maps of the 3 AMI groups, as well as separate MRI and PET images, are displayed in Figure 3. AMI scar size was significantly reduced after 1 month by both CDCs (13.2% ± 2.8%) and apoCDCs (13.1% ± 7.4%) as compared to untreated AMI animals (21.4% ± 2.3%) (Figure 3C). LV EF was improved compared to untreated AMI but failed to reach statistical significance (p = 0.13; CDC: 43.5% ± 7.1%, apoCDC: 45.8 ± 8.6, and untreated AMI: 39.8% ± 6.1%). 3-day LV end-diastolic volume (EDV) and end-systolic volume (ESV) were similar in all groups (Figure S6), but they were lower at the 1-month follow-up in both CDC and apoCDC groups, as compared to untreated AMI group. After normalization to body weight, LV EDV and ESV were both significantly reduced in apoCDC compared to controls and CDC (Figure 3C). Infarct size decreased significantly both in apoCDC (−6.7% ± 6.8%) and CDC (−8.4% ± 6.7%) as compared to the untreated AMI group (1.1% ± 6.7%) between 3 days and 1 month. LV EF increased in apoCDC (5.9% ± 6.3%) and CDC (4.0% ± 2.2%) but not the untreated AMI groups (−1.3% ± 7.7%); differences between the groups were not statistically significant.

**Figure 4:**
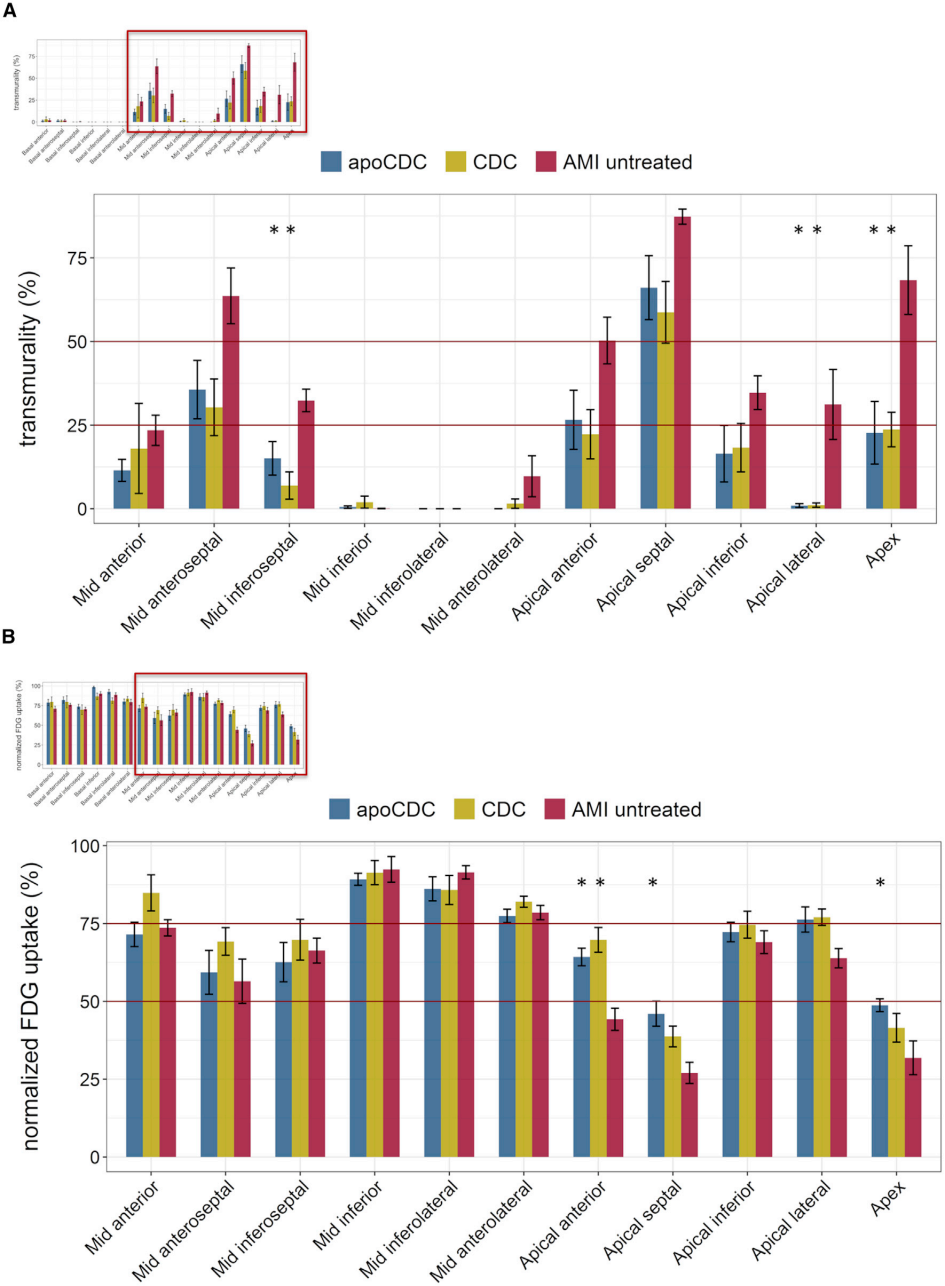
Segmental Analysis of Transmurality and [^18^F]FDG Uptake of Infarcted Porcine Hearts, 1 Month after Treatment with apoCDCs, CDCs, or Saline Control (A) Significantly lower transmurality was detected in mid-anteroseptal, inferoseptal, and apical segments in both CDC and apoCDC groups than in the untreated control animals. (B) Significantly higher FDG uptake was found in apical-anterior, apical-septal, and apex regions in apoCDC-treated animals and in the apical-anterior segment in CDC-treated animals compared to untreated controls. Data are mean ± SEM. ∗p < 0.05 (ANOVA with Bonferroni post hoc) compared to AMI untreated.

The average transmurality of the scar region was reduced when comparing the entire heart and the AMI segments. Segmental transmurality was significantly lower in mid anteroseptal, inferoseptal, and apical segments, both in CDC and apoCDC groups compared to untreated AMI groups (Figure 4A).

Standardized [^18^F]FDG tracer uptake was significantly higher in the apico-septal and apico-anterior and apex regions in the apoCDC animals as compared to untreated AMI pigs, whereas the CDC group showed a significantly higher uptake in the apico-anterior region (Figure 4B).

**Figure 5:**
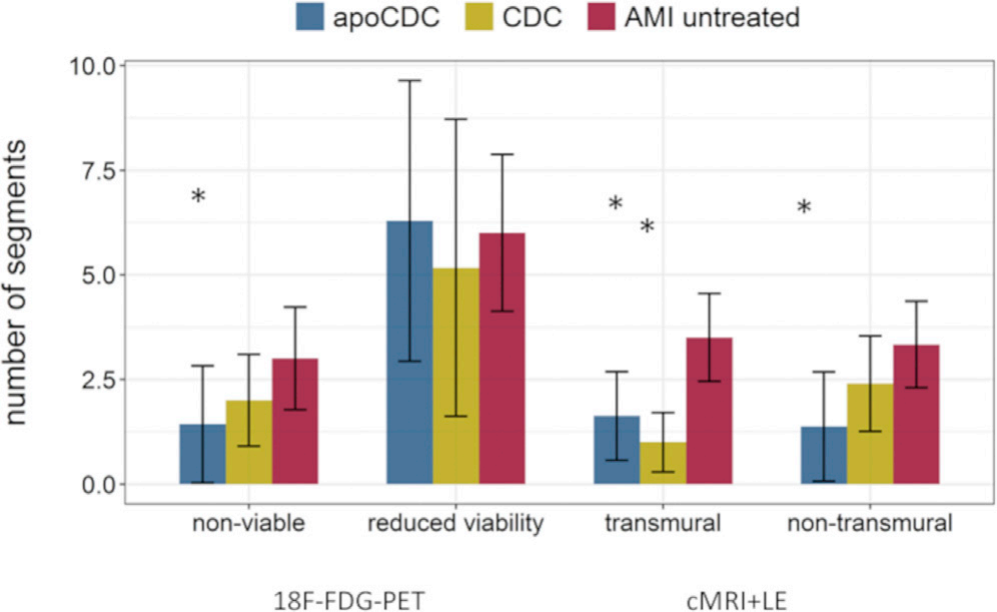
Number of Heart Segments in Different Groups with Reduced (50%–75%) or Lack of Viability (<50%) Assessed by [^18^F]FDG-PET-MRI and Number of Segments with Transmurality >50% and Nontransmural Scar between 25% and 50% Assessed by Cardiac MRI The number of transmural and nontransmural segments was lower after apoCDC treatment, with fewer transmural segments after CDC treatment compared to controls. Data are mean ± SEM. ∗p < 0.05 (ANOVA with Bonferroni post hoc) compared to AMI untreated.

APOSEC preconditioning led to a significantly lower number of transmural and nontransmural MRI-derived segments, whereas CDC treatment resulted in a significantly lower number of transmural segments in MRI, compared to the untreated AMI group (Figure 5). The composite number of nonviable segments and segments with reduced viability was significantly lower in both apoCDC (6.1 ± 1.6, p < 0.001) and CDC (6.3 ± 3.1, p = 0.01) compared to untreated AMI animals (13.0 ± 4.3). This highlights the higher sensitivity of [^18^F]FDG-PET imaging in assessment of cardiac repair.

After pooling segmental PET uptake and MRI transmurality data of all segments of all animals, [^18^F]FDG tracer uptake showed a significant negative correlation with infarct transmurality only at 1 month, indicating a mismatch between segmental transmurality and viability data early (3 days) after reperfused AMI, probably due to myocardial stunning or enhanced infiltration of inflammatory cells (Figure S4).

The 30-day right-ventricle (RV) MRI results are shown in Figure S5, with significant (p < 0.05) difference between sham control and all AMI groups regarding RV EF, which might be explained by the smaller scar size of the mid- and apical-interventricular septum. The 3-day scar and LV and RV data are shown in Figures S6 and S7, displaying no statistical difference among the 3 AMI groups at this time point.

### Effect of Regenerative Treatment on Gene and miRNA Expression in AMI, Border, and Remote Myocardium

**Figure 6:**
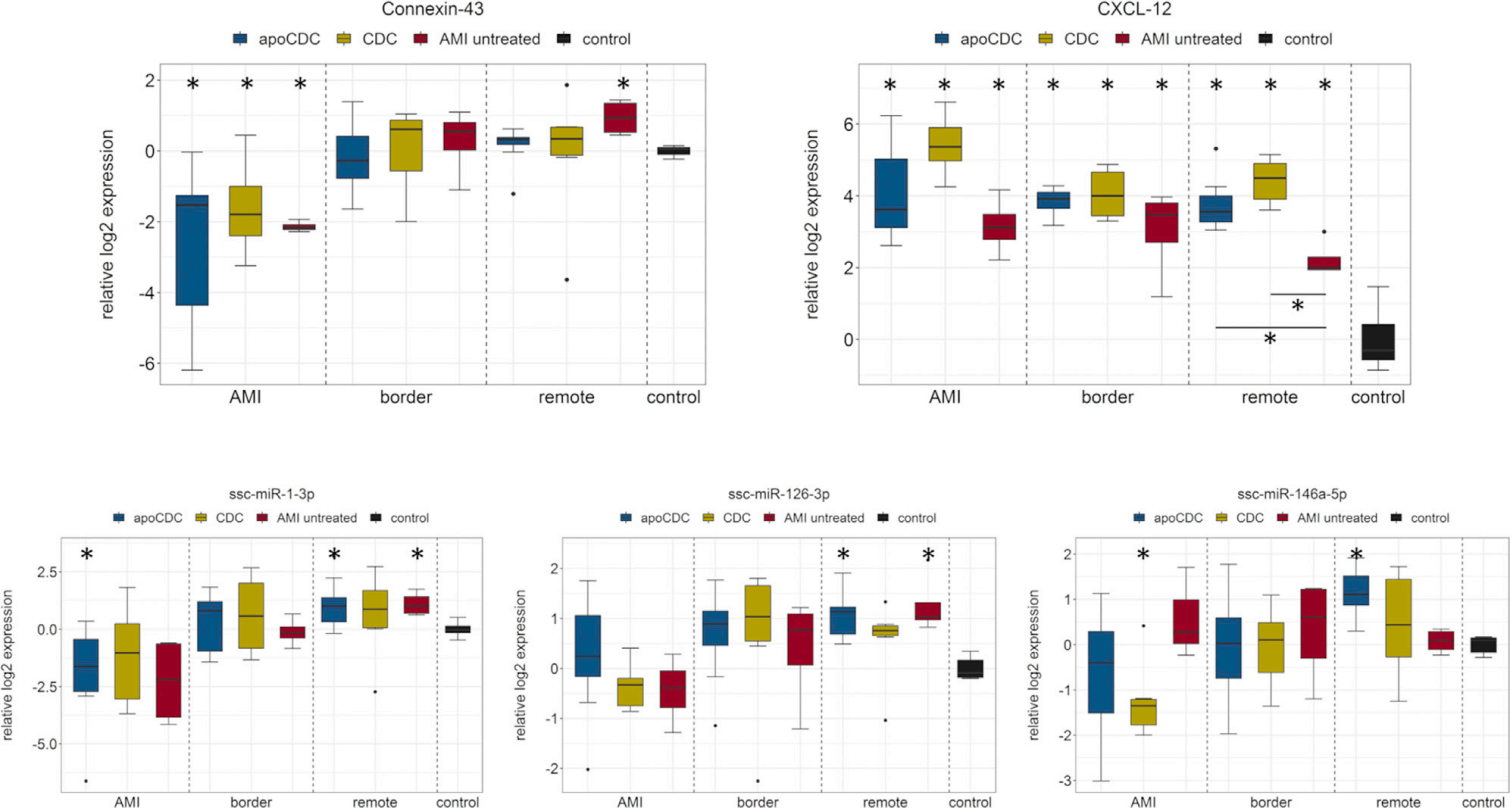
Analysis of Gene and miRNA Expression in Ischemic (AMI), Border, and Remote LV of Treated Pigs 1 Month after AMI and Receiving Stem Cell Treatment Control represents healthy (non-AMI) heart tissue. ∗p < 0.05 (ANOVA with Bonferroni post hoc) compared to healthy animals; compared to untreated where indicated.

We separately analyzed gene expressions in the myocardium directly affected by infarction (AMI), the adjacent area (border), and unaffected (remote) tissue to examine molecular mechanisms of regeneration in distinct areas. Hearts were explanted 1 month after infarction and treatment and used for analyzing tissue expression of selected genes and miRNAs to detect long-term effects of the distinct treatments. In our experiment, connexin 43 (*CX43*, gap junction protein 1 [*GJA1*]) expression was significantly lower after AMI compared to healthy animals, and both CDC groups showed a restoration of *CX43* expression in the AMI zone (Figure 6).

The CXCL12 (SDF-1)/CXCR4 axis is instrumental for stem and progenitor recruitment after MI. Our results show a marked increase of *CXCL12* expression in response to AMI in the infarct, border, and remote zones. Particularly in the infarct zone, expression was further enhanced in the apoCDC and CDC groups.

The cardiac-specific microRNA-1 (miR-1) has been shown to be downregulated in areas of acute infarction and upregulated in noninfarcted areas in human autopsy samples.^18^^,^^19^ The regulation of miR-1 is known to be triggered shortly after the onset of infarction. We found a similar pattern in nontreated infarcted pig hearts 1 month after MI compared to the myocardium of control animals and a nonsignificant trend toward increased miR-1 levels in infarcted myocardium after treatment with CDCs or apoCDCs. The expressional levels of miR-1 and its known molecular target *CX43*^20^ are inversely correlated.

In treated and untreated pig hearts, miR-126 expression was reduced 1 month after AMI in the infarcted areas but largely unchanged in the border and remote zones. Regenerative treatments did not result in significant expressional changes in comparison to untreated animals. miR-146a showed minor changes in expression in treated animals compared to both untreated infarcted pigs and sham-operated control pigs.

## Discussion

In this work, we show for the first time the following: that (1) preconditioning of cardiac allogeneic CDCs with APOSEC preserves the stem-cell properties and limits the production of proteins involved in cross-differentiation toward a myofibroblast-like phenotype; (2) cardiac MRI + LE might be insensitive to show detailed regenerative effects of cardiac-regenerative stem cells; (3) [^18^F]FDG-PET imaging is more attractive to demonstrate preserved viability of the ischemic-injured myocytes, being subjected to paracrine-reparative influence of stem cells; (4) simultaneous acquisition of [^18^F]FDG and MRI images analyzed with custom-made software provides supplementary information on segmental viability and transmurality; and (5) preconditioned CDCs with paracrine stimuli lead to smaller areas with transmural and nonviable myocardium and an improvement in LV EDV (LVEDV) and ESV relative to body weight in a preclinical model of reperfused AMI.

### apoCDC

APOSEC has anti-inflammatory and cell-protective effects and has been extensively characterized *in vitro* and *in vivo*.^13^^,^^17^^,^^21^^,^^22^ Clinical development for a topical application for wound healing in diabetic foot ulcers has been initiated. Direct induction of cardiac repair by APOSEC after AMI in pigs was evaluated earlier.^13^^,^^14^ Here, we have investigated the effect of *in vitro* conditioning of CDCs with the cell-protective, prosurvival, and proangiogenic APOSEC. *In vitro* culturing of CDCs in the presence of APOSEC significantly altered the molecular properties of CDCs in terms of proteome secretion and selected markers of gene expression. Overall, CDCs under standard, serum-free cell-culture conditions increasingly secreted ECM proteins, indicating a certain degree of cross-differentiation toward a myofibroblast-like phenotype. In contrast, APOSEC preserves the characteristics of CDCs *in vitro* for at least 72 h. The use of APOSEC (or similar medium additives) may be useful for *in vitro* propagation of cardiac progenitor cells and for modulating and enhancing therapeutic effects.

### Simultaneous Assessment of Cardiac Function and Myocardial Viability

Both CDCs and apoCDCs resulted in smaller scar sizes, as determined by cardiac MRI, 1 month after the onset of infarction. We have previously shown that intravenous application of APOSEC in small and large animal AMI significantly reduced scar size and improved LV EF.^12^ In previous experiments, intracoronary administration of APOSEC in the reperfusion phase of AMI failed to improve effects of intravenous APOSEC infusion (unpublished data). Therefore, we did not compare the *in vivo* effects of intracoronary CDCs and APOSEC in this study but investigated the APOSEC-enhanced CDC effects on myocardial performance. The reduction of the LV EF after 1 month was mitigated by cell treatment, but differences between CDC/apoCDC and untreated animals failed to reach significance. This finding is in line with previous investigations in large animal models and in clinical trials, showing that stem-cell transplantation fails to result in a clinically meaningful improvement of heart function in most cases.^23^ In order to gain further insight into cell-therapy mechanism, we used hybrid cardiac MRI + LE with [^18^F]FDG-PET imaging by a clinical camera. Increased uptake of the PET tracer in apical anterior and septal segments indicates beneficial effects of cell treatment and in a higher extent after CDCs were preconditioned with APOSEC. We analyzed both images by using the 17-segment model and revealed small but significant changes in ischemic-injured cell viability and transmurality in the cell-treated groups. This is the first report on simultaneous quantitative assessment of hybrid PET-MRI images.^24^ The discrepancy between viability assessment by [^18^F]FDG-PET and MRI might reflect a higher sensitivity of PET tracer imaging. Additionally, in the acute and subacute phase of AMI, the increased [^18^F]FDG uptake might be caused by a detection of infiltrating cells of the immune system or progenitor cells, which enhances glucose uptake.^25^ However, infiltration of activated immune cells is regarded to be negligible 1 month after MI. Several advantages and drawbacks, as well as challenges associated with PET and MRI, respectively (Table 2), are well known.^26^ For assessment of therapeutic values of cardiac repair modalities, [^18^F]FDG-PET and hybrid PET-MRI systems may be superior for precise assessment of beneficial effects.

**Table 2:** Short Comparison of Imaging Techniques for Assessment of Myocardial Infarction

	[^18^F]FDG-Positron Emission Tomography (PET)	Magnetic Resonance Imaging (MRI)
Advantages	high sensitivity, high accuracy for viability detection	high spatial resolution, high accuracy for scar detection
Disadvantages	exposure to ionizing radiation, lower spatial resolution	lower specificity of LGE
Provided information in heart imaging	myocardial viability, enhanced glucose uptake in inflammatory cardiac diseases enhanced glucose uptake in hibernating myocardium	myocardial function parameter: systolic and also diastolic, infarct size, myocardial edema, area at risk
Limitations	interference of inflammation, cost, availability restricted to specialized centers	interference by medical devices and metal implants

LGE, late gadolinium enhancement.

Overall, these results are in line with the current knowledge of cardiac regeneration by progenitor cells in large animal models and the clinical setting. In our model, apoCDC failed to demonstrate a significant increase in LV EF, but it improved LVEDV and LV ESV (LVESV) relative to body weight and increased the viability of the myocardial cells and decreased infarct size and the number of segments with reduced or nonviable cardiomyocytes, keeping the door open for further recovery. The effects on heart function observed in our study may be improved by further optimizing preconditioning of CDCs through improving APOSEC concentration or composition. Previous studies have shown that individual APOSEC components or paracrine factors derived from distinct PBMC subsets fail to fully replicate functional APOSEC effects. In addition to the use as an active pharmaceutical ingredient (API), our data show that APOSEC may hold promise for enhancing properties or cardiac progenitor cells.

1 month after cell treatment, the differences in expression of the investigated genes and miRNAs were small, and no distinct effects of the treatment were identified. Of the investigated molecular markers, the tissue expression of *CXCL12* was increased significantly in all groups compared to sham-operated animals, indicating a prominent role of *CXCL12* after infarct. *CX43* expression was reduced in infarcted areas but not the border or remote zones. We found a strong inverse correlation between miR-1 and Cx43 expressions, confirming the effect of miR-1 on its gene target *CX43*.

### Limitations

This preclinical study was performed in healthy young animals, which lack comorbidities frequently observed in human patients, such as hypertension, atherosclerosis, diabetes, etc. Influences of these on the scar size and LV dilation and remodeling are therefore disregarded in this study.

A 1-month follow-up period (FUP) shows the scar size developed after ischemic injury. For development of chronic ischemic heart failure and more detailed investigation of functional consequences of the stem-cell therapy, a longer FUP of at least 2 or 3 months would be required. We were thus unable to detect possible longer-term functional improvements by either CDCs or apoCDCs in this study.

The animals did not receive standard medical treatment to prevent LV dilation and remodeling (e.g., angiotensin-converting enzyme [ACE] inhibitors, angiotensin receptor blockers [ARBs], or beta-blockers). However, they were treated with the dual antiplatelet therapies. The general health status of the animals was monitored on a daily basis, but blood pressure, heart rate, electrocardiogram (ECG), and laboratory values for a medical therapy guide were checked at the final follow-up examination only. Any of the above-mentioned information would have required anesthesia, with the risk of anesthetic complication of the animals with AMI.

### Conclusion

We have identified protecting and beneficial effects of APOSEC on CDCs in *in vitro* cell culture. Intracoronary infusion of apoCDCs in the reperfusion phase of AMI led to significantly better myocardial viability and lower transmurality in the infarcted area, demonstrated by PET-MRI imaging.

### Translational Outlook

Although cell preconditioning with APOSEC failed to translate into robust enhancement of cardiac functional outcomes in a large-animal AMI model, our data demonstrate an essential influence of cell-culture conditions on the paracrine signature of stem cells, and further optimization is a viable strategy for rational improvement of stem cells in cardiac applications. Hybrid PET-MRI imaging appears to be more sensitive to assess cardiac repair therapy effects. Together with reduced infarct scar sizes by CDCs and apoCDCs, these data might constitute further improvement of the paracrine-stimulator cell-based therapy.

## Materials and Methods

### CDC Culture

Porcine allogeneic CDCs were isolated as described.^8^ CDCs were resuspended (1.25 × 10^6^/mL for a total dose of 12.5 × 10^6^) in CryoStor CS10 (BioLife Solutions) in Cryobags (PL07 PermaLife Bags; OriGen Biomedical), placed directly in a CryoMed controlled-rate freezer and then transferred to liquid nitrogen. Cells were prepared at the Smidt Heart Institute, shipped to the Medical University of Vienna, and thawed on site for *in vitro* preconditioning and *in vivo* experiments.

### APOSEC Preparation

Porcine APOSEC was obtained as described.^27^ The obtained solution was sterile filtered, frozen, and lyophilized overnight. Lyophilized APOSEC aliquots were stored at −80°C.

### *In Vitro* Effect of APOSEC on CDCs—Proteomics

Porcine CDCs were cultured in growth medium (Iscove’s modified Dulbecco’s medium [IMDM], supplemented with 10% fetal calf serum [FCS] and penicillin/streptomycin) in 5% CO_2_ at 37°C. Cells were incubated with serum-free medium (4 mL), with or without APOSEC, as detailed in the Supplemental Information. For proteomic analysis, the proteins of the supernatant were subjected to label-free shotgun proteomics on a liquid chromatography (LC)/MS system. Quantitative data were assessed by MACSQuant and processed in R,^28^ with the limma package^29^ and clusterProfiler.^30^ Gene expression was analyzed by qRT-PCR, and cytokine concentrations were analyzed using commercial ELISA kits (porcine VEGF and CXCL12/SDF-1; Sigma-Aldrich), as detailed in the Supplemental Information.

### APOSEC Pretreatment of CDCs for *In Vivo* Application

For APOSEC conditioning, CDCs were thawed, seeded in 300 cm^2^ flasks (1.6 × 10^7^ cells per flask), and cultured in growth medium (IMDM, 20% FBS, supplemented with gentamicin and β-mercaptoethanol) for 24 h. The next day, growth medium was replaced with fresh medium containing APOSEC (in a final concentration of APOSEC derived from 10.0 × 10^6^ cells/mL), and cells were incubated for further 48 h. The supernatant was removed, and cells were washed with PBS, trypsinized, and resuspended in CryoStor CS10 and passed repeatedly through a gauge. Cell numbers were counted, and the cell suspension was diluted to 1.7 × 10^6^ cells/mL (7 mL for one dose). Upon thawing, 1 mL 100 IU/mL heparin and 0.1 mL nitroglycerin (50 μg/mL) were added to the cell suspensions.

### Animal Experiments

All applicable international, national, and/or institutional guidelines for the care and use of animals were followed. All procedures were performed with the approval of the local Ethical Committee of the Experimental Animal Care Committee of the University of Kaposvár, Hungary (approval reference: SOI/31/1220-6/2015 (KA-1777)), conforming to the Guide for the Care and Use of Laboratory Animals published by the US National Institutes of Health. The animals were randomly assigned to groups AMI (n = 22) or control (n = 5) (Figure 1). The pigs in AMI groups underwent a reperfused AMI protocol, whereas the control animals received only the catheterization. One pig died during the AMI protocol. All other animals completed the study period and were included in the analyses. Investigators were blinded for PET/MRI imaging and downstream analyses.

Domestic pigs (female, 15 kg, n = 26) were fasted overnight and were sedated with 12 mg/kg ketamine hydrochloride, 0.04 mg/kg atropine, and 1.0 mg/kg xylazine, followed by intratracheal intubation. The anesthesia was then continued with 1.5–2.5 vol% isoflurane, 1.6–1.8 vol% O_2_, and 0.5 vol% N_2_O. During anesthesia, continuous monitoring of the O_2_ saturation and ECG were performed. After surgical preparation of the right arteria femoralis, a 6F introducer (Medtronic, Minneapolis, MN, USA) was placed. After administration of 200 IU/kg unfractionated heparin intra-arterially, 6F right coronary guiding catheters were introduced into the left and right coronary ostium to perform selective angiography of the left and right coronary arteries.

In the AMI pigs, the mid-left anterior descending artery (LAD) was occluded by inflating a balloon (2.75 mm in diameter, 9–12 mm long) at 5 atm for 90 min, followed by reperfusion via balloon deflation. Immediately after induction of reperfusion, an additional dose of 2,000 IU heparin was administered in order to decrease microvascular obstruction. 15 min after a control angiography (to prove the patency of the infarct-related artery), an over-the-wire balloon was inserted distal to the previously occluded site, and the CDCs (10^7^ cells, suspended in 20 mL saline with 2,000 IU heparin and 0.1 mL nitroglycerin), with (group apoCDC, n = 8) or without APOSEC preconditioning (group CDC, n = 6) or saline solution (group AMI-untreated, n = 7), were infused (1 mL/min) into the LAD by selective intracoronary infusion. After hemodynamic stabilization, the pigs were allowed to recover.

All pigs received a standard loading dose of clopidogrel (300 mg) and aspirin (250 mg), 1 day prior to the procedure, and the standard postinfarction daily medical treatment of 100 mg aspirin and 75 mg clopidogrel. Routine blood parameters (electrolyte, blood sugar, hematologic parameter, kidney, liver function) were monitored before AMI, after reperfusion, and after the respective treatment, furthermore at 3 days and 30 days post-AMI. At the 3-day FUP, cardiac [^18^F]FDG-PET-MRI with LE was performed to measure LV performance and to identify the area at risk. This was repeated at the 30-day FUP, accompanied by control invasive hemodynamic assessments.

### Follow-Up Investigations

After overnight fasting, all pigs in AMI groups underwent hybrid [^18^F]FDG-PET-MRI with LE, whereas control animals received MRI + LE without PET, 3 and 30 days after the sham procedure, respectively. At the final 30 days follow-up, left-heart catheterization was performed under general anesthesia to ensure the patency of the infarct-related artery, followed by humane euthanasia by intravenous application of 10 mL saturated potassium chloride. The hearts were explanted, and myocardial samples from the infarcted border zone of AMI and remote posterior-wall myocardial areas were immediately cut and stored in RNAlater (QIAGEN, Germany). Myocardial samples from the mid-anterior region of the control animals were also stored.

### PET-MRI Imaging Acquisition

[^18^F]FDG-PET-MRI imaging was performed by using a hybrid PET/MRI system (Biograph mMR; Siemens Medical Solutions, Erlangen, Germany), allowing the simultaneous acquisition and analysis of PET and MRI data in a one-step mode. The cardiac MRI part of the acquisition has been performed by using a 3T MRI, whereas the PET scanner is built of lutetium oxyorthosilicate (LSO) crystals equipped with avalanche photodiodes.^31^

For the assessment of the cardiac function and the visualization of possible wall-motion abnormalities, multislice-multiphase cine imaging was performed in the long horizontal axis, as well as in the short axis view through the entire heart.

Myocardial function and scar size were assessed by MRI after an intravenous bolus of a contrast agent (0.05 mmol/kg gadobenate dimeglumine [MultiHance®, Bracco Imaging, Italy], 4 mL/s), with an automatic injector (Medrad Spectris, USA). During administration of the contrast agent with a power injector, time-resolved imaging of three slices in the short axis and one slice in the horizontal long axis was acquired to detect perfusion deficits. After a delay time of at least 10 min after contrast agent injection, LE imaging (late gadolinium enhancement [LGE]) was performed to assess myocardial scar size. Prior to the acquisition of the LE sequence, a scout for the assessment of the optimal inversion time (T1) was done. The LE imaging was performed in the short axis and the 4- and 3-chamber views.

For PET imaging, a list-mode ECG-gated PET scan in 3D mode was initiated 1 h after intravenous injection of 300 MBq [^18^F]FDG in fasted animals, according to an established protocol.^24^ Blood sugar, electrolyte, and kidney function were assessed prior to imaging acquisition. Emission data correction was done for dead time, randoms, scatter, and attenuation. A 3D attenuation-weighted ordered-subsets expectation maximization (AW-OSEM 3D)-iterative reconstruction algorithm with Gaussian smoothing at 4 mm full width at half maximum and a matrix size of 344 × 344 were used for image reconstruction. ECG-triggered long-axis (two- and four-chamber views) and short-axis images were acquired during breath hold.

### Comparative Semiquantitative Analysis of Myocardial Viability and Quantitative Assessment of LV Function by Hybrid PET-MRI Imaging

The EFs of both the LV and RV, as well as further functional parameters, including ESV, EDV, and stroke volume (SV), were calculated in a semi-automatic fashion based on the short-axis views and were related to the actual body weight (LV EDV index [EDVi], ESV index [ESVi], and SV index [SVi]). The infarcted myocardial mass was determined based on the LE MR images, and infarct size was calculated relative to the LV mass and expressed as percentage of the entire LV. The infarct-size measurement was based on a semi-automatic approach, with the hyperintense areas on MR images assessed 10 min after gadolinium contrast agent administration. Infarct sizes were determined using the MEAN + 2SD method, where MEAN refers to the mean intensity values of a remote (i.e., unaffected) myocardial segment, whereas the scar is defined as image pixels with higher intensity values than the sum of the mean plus two standard deviations of the unaffected myocardium. Volumetric MRI analysis and infarct-size measurements were performed using the freely available software Segment (Medviso AB, Lund, Sweden).^32^ For semiquantitative assessment of infarct transmurality, the LGE images were divided into 17 segments, according to the American Heart Association (AHA) recommendation of 17-segment analysis. Reference “no scar” zero point and 100% as transmural area of the highest intensity in the LGE areas of the remote posterior and infarcted areas were chosen, respectively, and the transmurality of other segments was related to these values. Transmural, reduced transmurality, and no transmurality of the segments were defined as >75%, 25%–75%, and <25%, respectively, of intensity.

Quantitative segmental analysis of the attenuation-corrected PET images were performed by using the acquisition 60 min after [^18^F]FDG tracer injection. In order to enable direct segmental comparison of PET and MRI images, an in-house software was developed, and we divided the myocardium to 17 segments. Segmental analysis was performed by overlaying the linear borders of a 17-segment LV model on the PET image. We visually controlled the location of the model outlines on the PET-visible LV and RV borders on all slices that comprised the heart volume from the apex to the level of the great vessels. Analysis was consistently restricted to slices between the apex and the basis on which the LV myocardium is seen in 360°. The optimal rotation of the 17-segment model around the apex-basis axis was determined by matching the mid-septal points in the image and the model. The endo- and epicardial borders were segmented based on the visual features of the PET image. Tracer uptake was calculated using a semiquantitative method. First, we calculated the average image intensity of a given LV segment in the bulls-eye plot tool of Segment. The standardized uptake value (%) of the tracer for each segment was calculated by normalizing their values to the “hottest” LV segment. Nonviable segments were defined as <50% segmental tracer uptake.^24^ For a more refined assessment, we additionally defined segments with 50%–75% [^18^F]FDG uptake as having reduced viability.^33^

For quality control, we have performed quality check and reproducibility measurements by analyzing selected 1-month follow-up [^18^F]FDG-PET-MRI images of ten randomly chosen animals, twice by the same observer (intraobserver) and by two independent observers (interobserver). Repeat measurements of the normalized FDG uptake of the 17 segments of the ten images resulted in a regression coefficient of variation of the repeated analyses between 0.87 and 0.92 (p < 0.001) (intraobserver variability) and 0.81 and 0.89 (p < 0.001) (interobserver variability).

### Statistical Analyses

Statistical significance was calculated by comparing the groups using ANOVA with Bonferroni post hoc analyses in SPSS statistics, version 21.0. Bivariate correlations were calculated using Pearson coefficient. Statistical significance of proteomics data was calculated using limma with voom transformation.^29^ PET-MRI and gene-expression analyses were performed by blinded observers.

## Author Contributions

Conception and Design, Collection and/or Assembly of Data, Data Analysis and Interpretation, Manuscript Writing, J.W.; Collection and/or Assembly of Data, Data Analysis and Interpretation, D.L. and J.M.-T.; Collection and/or Assembly of Data, K.Z., A.G., N.P., D.T., C.M., A.S., M.R., and E.H.; Data Analysis and Interpretation, A.J. and Z.S.; Provision of Study Material or Patients, J.D. and M.Z.; Conception and Design, Administrative Support, Provision of Study Material or Patients, Manuscript Writing, Final Approval of Manuscript, H.J.A.; Conception and Design, Administrative Support, Provision of Study Material or Patients, Final Approval of Manuscript, E.M.; Conception and Design, Financial Support, Administrative Support, Collection and/or Assembly of Data, Data Analysis and Interpretation, Manuscript Writing, Final Approval of Manuscript, M.G.

## Conflicts of Interest

The APOSEC platform technology is patented, and financial interest is claimed by the Medical University and APOSCIENCE AG. APOSCIENCE AG holds patents related to this work (EP 20080450198, EP 20080450, and EP 17209165). H.J.A. is shareholder of APOSCIENCE AG. The other authors declare no competing interests.

## Supplemental Information

Document S1. Figures S1–S8, Table S3, and Methods S1

Table S1. Quantitative Proteomics Data of CDC in Cell Culture with and without Aposec. Values are reported as arbitrary abundance derived from MacsQuant software as mean±s.d (for three biological replicates, each the result of three technical replicates).

Table S2. Quantitative Proteomics Data of CDC in Cell Culture with and without Aposec, Listed According to the Protein Clusters in Figure 2. Values are reported as arbitrary abundance derived from MacsQuant software as mean±s.d (for three biological replicates, each the result of three technical replicates).

Document S2. Article plus Supplemental Information
