# Supplementary material for: Quantitative Hybrid Cardiac [18F]FDG-PET-MRI Images for Assessment of Cardiac Repair by Preconditioned Cardiosphere-Derived Cells
Source: Mol Ther Methods Clin Dev. 2020 Jun 15;18:354–66. doi: 10.1016/j.omtm.2020.06.008 (PMC7341058; doi:10.1016/j.omtm.2020.06.008)
Supplement: Document S1. Figures S1–S8, Table S3, and Methods S1 [file mmc1.pdf]

OMTM, Volume *18*

Supplemental Information

Quantitative Hybrid Cardiac [^{18}F]FDG-PET-MRI Images for Assessment of Cardiac Repair by Preconditioned Cardiosphere-Derived Cells

Johannes Winkler, Dominika Lukovic, Julia Mester-Tonczar, Katrin Zlabinger, Alfred Gugerell, Noemi Pavo, András Jakab, Zsuzsanna Szankai, Denise Traxler, Claudia Müller, Andreas Spannbauer, Martin Riesenhuber, Ena Hašimbegović, James Dawkins, Matthias Zimmermann, Hendrik J. Ankersmit, Eduardo Marbán, and Mariann Gyöngyösi

Supplemental Figures

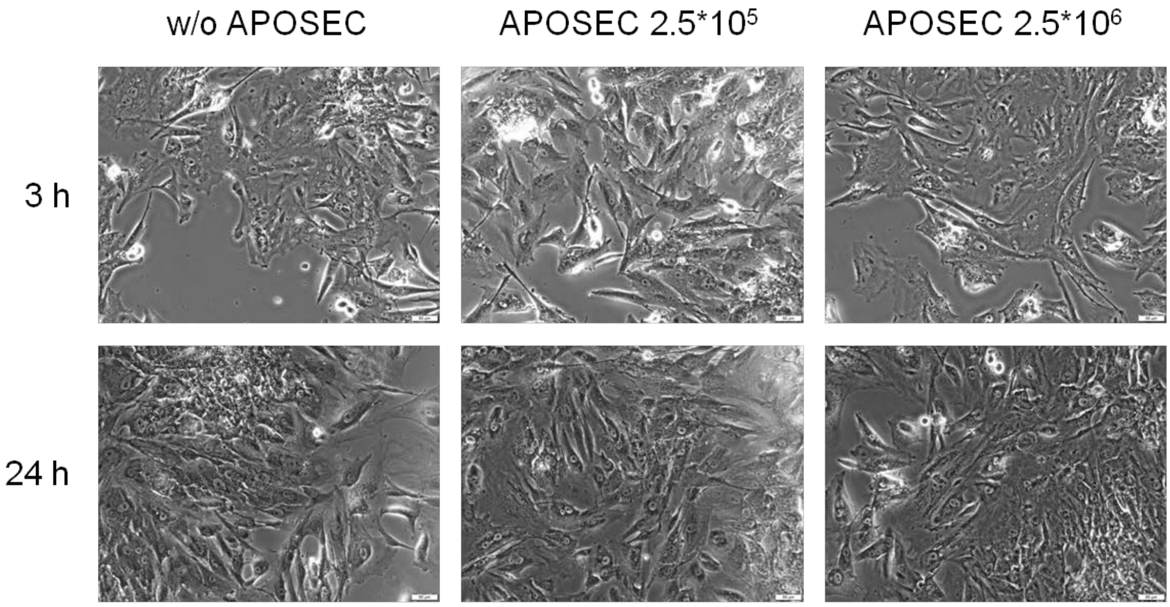

Figure S1. CDC cells after incubation with/without Aposec.

CDC cells after 24h incubation in standard growth medium, with Aposec derived from $2.5x10^5$ cells or from $2.5x10^6$ cells. APOSEC treatment had no effect on cell viability and proliferation.

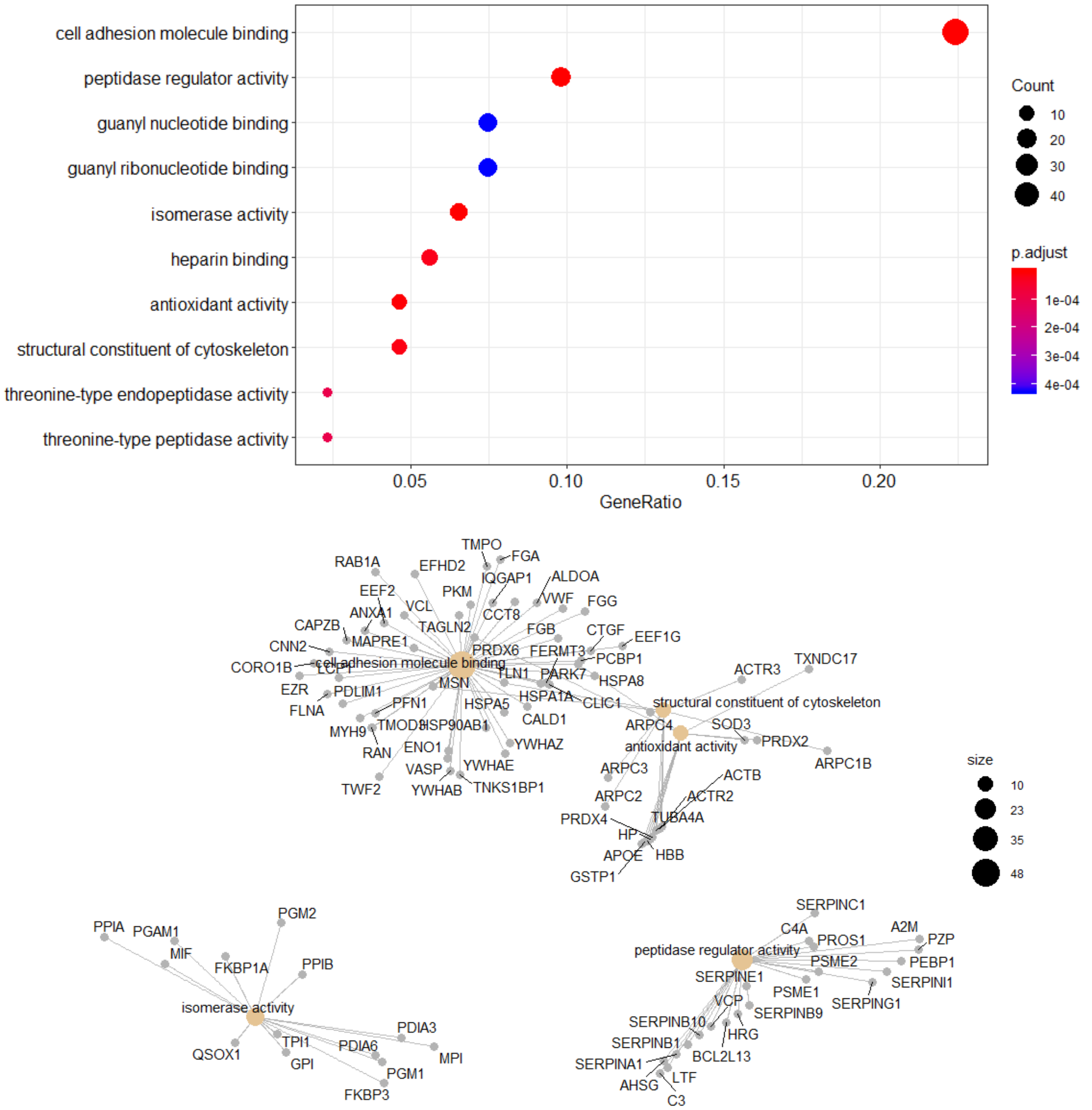

Figure S2. Gene Ontology (GO) analysis of porcine APOSEC protein composition (molecular function).

The upper panel shows gene ratios with adjusted p values, the lower panel the protein networks of the major GO terms.

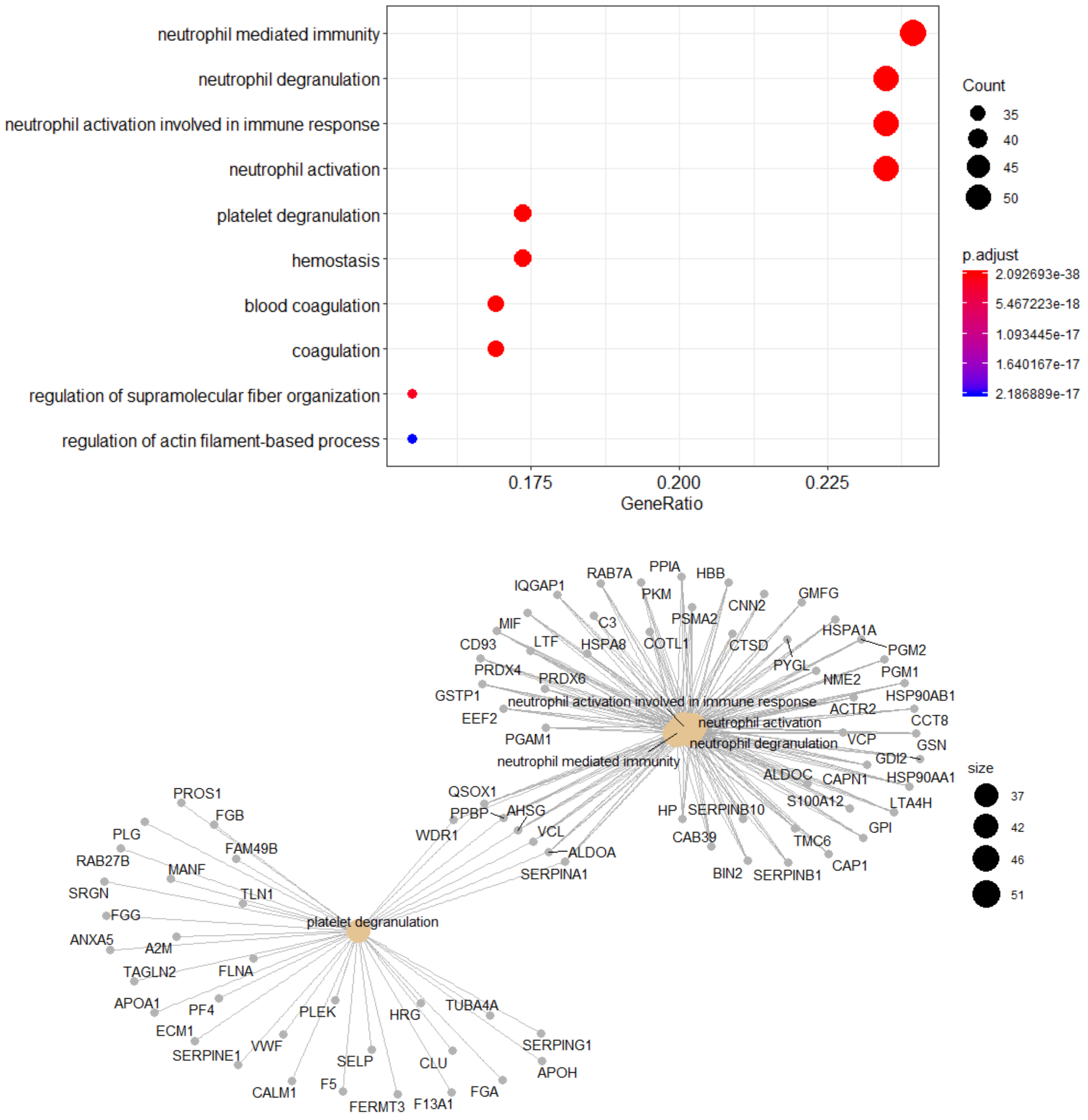

Figure S3. Gene Ontology (GO) analysis of porcine APOSEC protein composition (molecular function).

The upper panel shows gene ratios with adjusted p values, the lower panel the protein networks of the major GO terms.

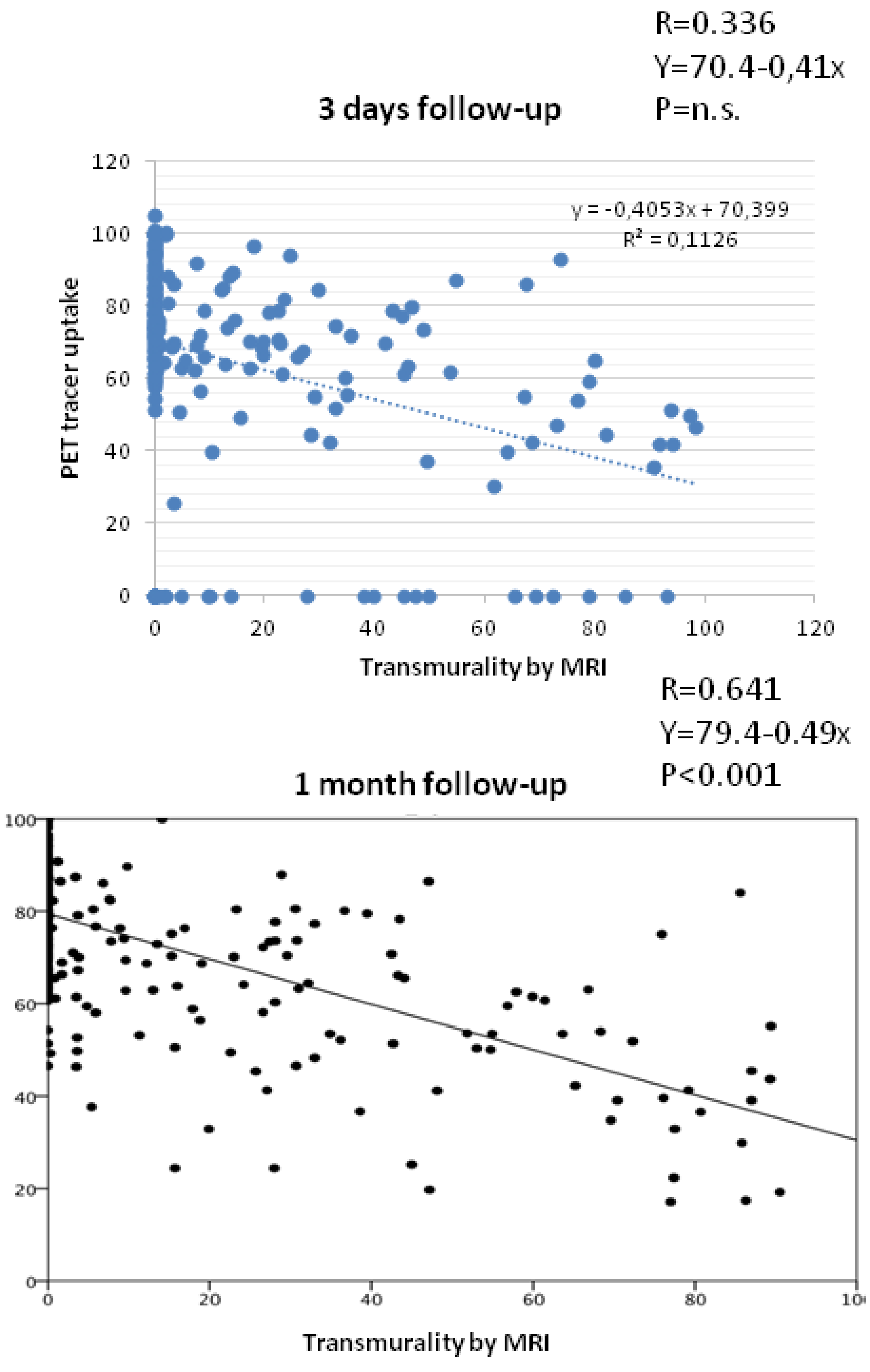

Figure S4. Segmental correlation between MRI-derived transmurality and [^{18}F]FDG-derived viability at 3 days and 1 month after reperfused acute myocardial infarction.

Pooled segmental data of all animals.

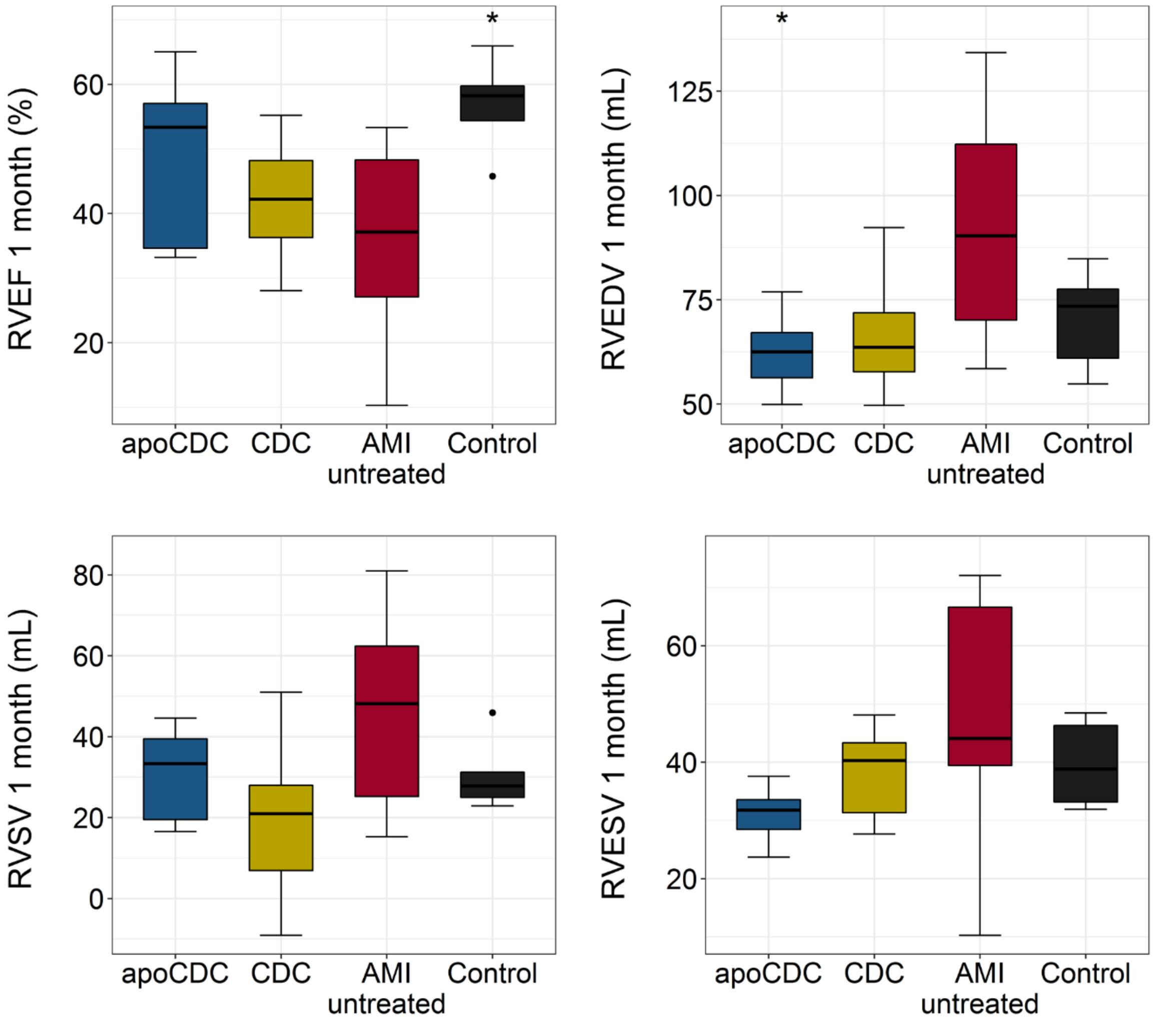

Figure S5. MRI-derived right ventricular (RV) data at 1 month after reperfused AMI and treatment with apoCDC or CDC or saline, and sham-operated animals.

ApoCDC, n=8; CDC, n=6; AMI untreated, n=7; control, n=5.

*p<0.05 between Control and all AMI groups

EF: ejection fraction, EDV: end-diastolic volume, ESV: end-systolic volume, SV: stroke volume

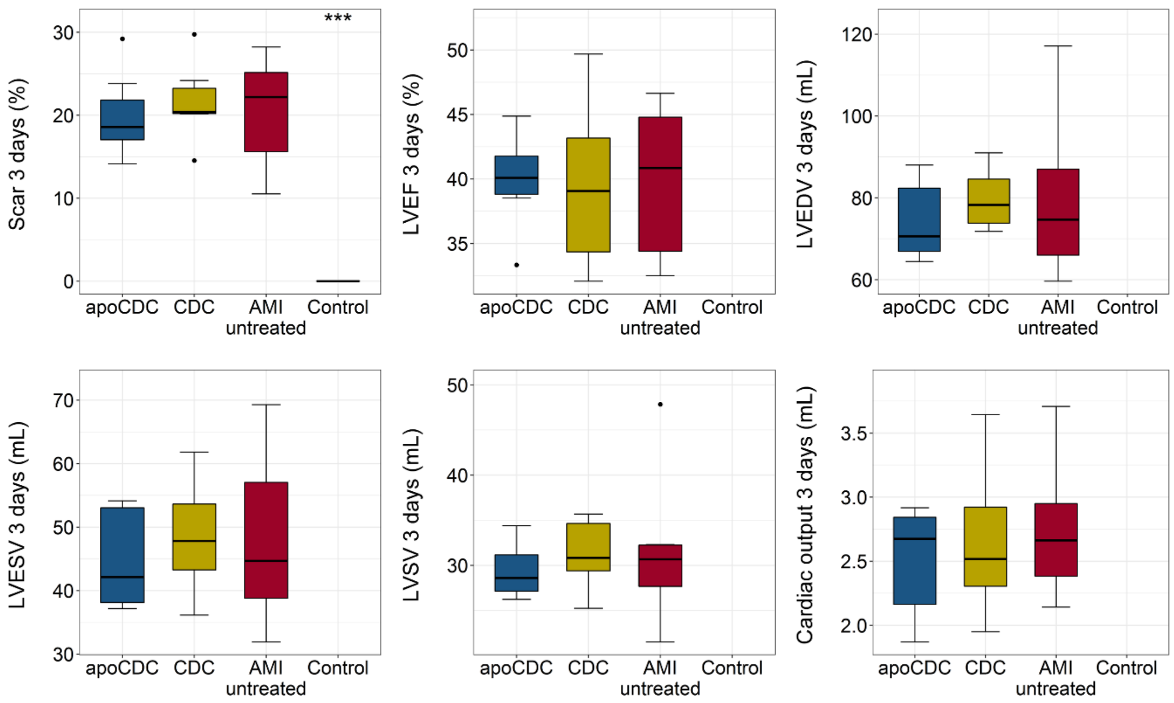

Figure S6. MRI-derived left ventricular (LV) data at 3 days after reperfused AMI and treatment with apoCDC or CDC or saline, and sham-operated animals.

ApoCDC, n=8; CDC, n=6; AMI untreated, n=7; control, n=5.

*p<0.05 between Control and all AMI groups

EF: ejection fraction, EDV: end-diastolic volume, ESV: end-systolic volume, SV: stroke volume

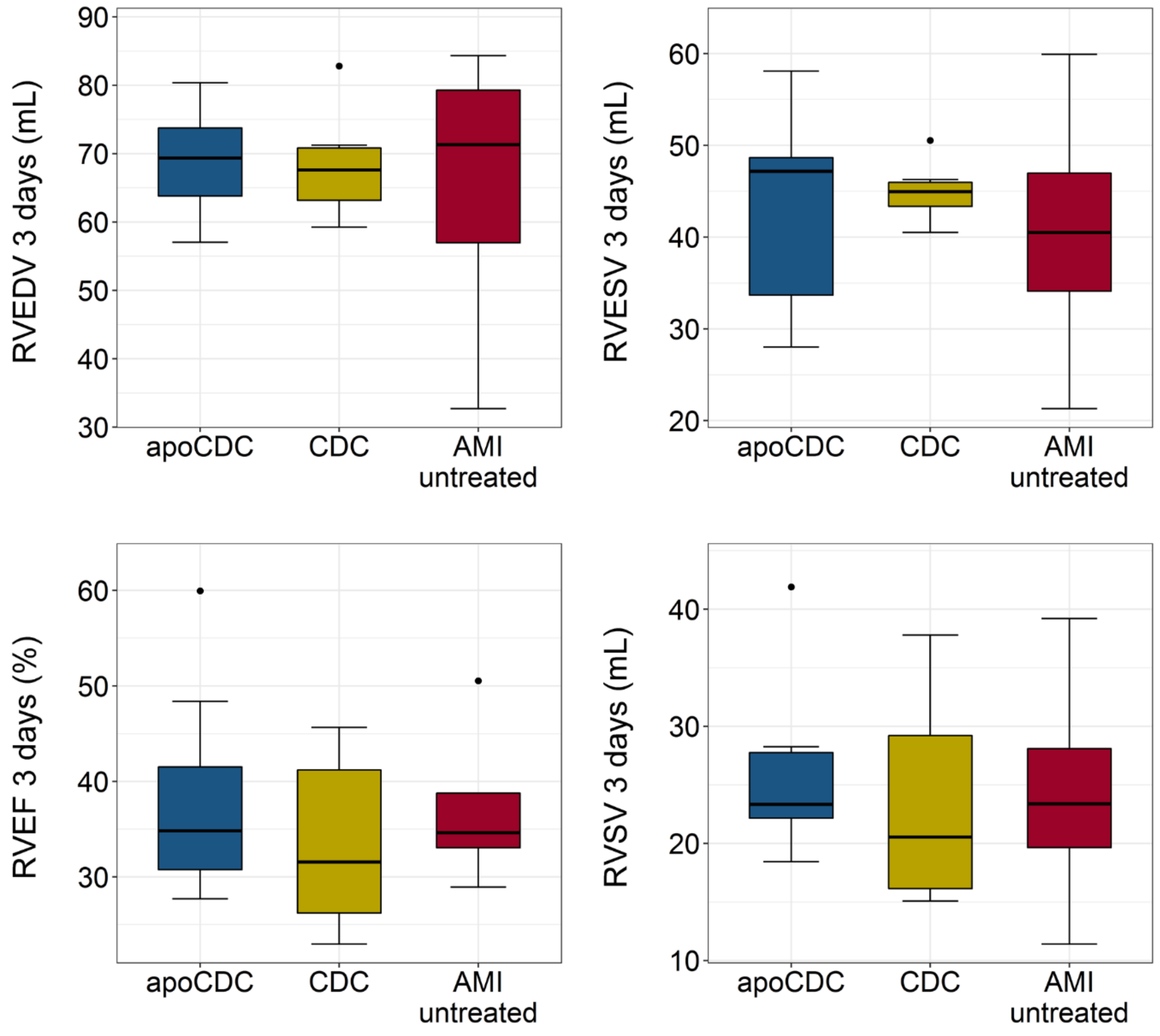

Figure S7. MRI-derived right ventricular (RV) data at 3 days after reperfused AMI and treatment with apoCDC or CDC or saline, and sham-operated animals.

ApoCDC, n=8; CDC, n=6; AMI untreated, n=7; control, n=5.

EF: ejection fraction, EDV: end-diastolic volume, ESV: end-systolic volume, SV: stroke volume

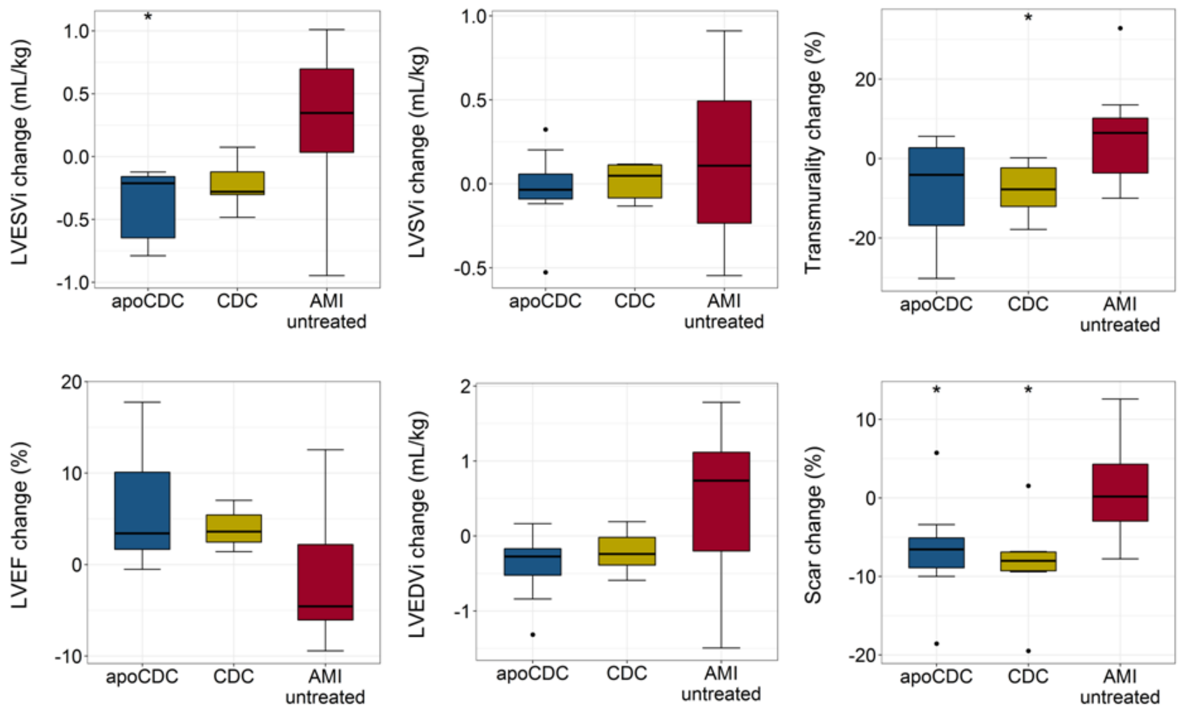

Figure S8. Changes of MRI-derived data between 3days and 1 month after AMI and treatment.

*p<0.05 compared to AMI untreated

Biological process

ID	Description	Adjusted p-value	Gene Count
GO:0030198	extracellular matrix organization	1.89E-29	34
GO:0031589	cell-substrate adhesion	4.99E-09	17
GO:0002576	platelet degranulation	8.46E-08	11
GO:0060351	cartilage development involved in endochondral bone morphogenesis	2.90E-06	7
GO:0006614	SRP-dependent cotranslational protein targeting to membrane	2.94E-06	9
GO:0035966	response to topologically incorrect protein	2.94E-06	11
GO:0070972	protein localization to endoplasmic reticulum	2.94E-06	10
GO:0002446	neutrophil mediated immunity	3.84E-06	16

Molecular function

ID	Description	Adjusted p-value	Gene Count
GO:0005201	extracellular matrix structural constituent	1.81E-33	29
GO:0050839	cell adhesion molecule binding	9.21E-16	26
GO:0005178	integrin binding	9.21E-10	12
GO:0005539	glycosaminoglycan binding	3.54E-09	14
GO:0005518	collagen binding	1.13E-08	9
GO:0023026	MHC class II protein complex binding	1.12E-06	5
GO:0050840	extracellular matrix binding	1.40E-06	7
GO:0008201	heparin binding	1.49E-06	10

Table S3. Top gene ontology terms for proteins secreted by CDCs.

Supplemental Methods

Gene expression analyses

For RNA isolation, tissue samples of around 25 mg were cut to small pieces, transferred to 700 µl Qiazol solution and homogenized using a Precellys system (Peqlab, Germany). Total RNA including small RNA was extracted using the miRNeasy Mini Kit (Qiagen, Germany) on a Qiacube according to the manufacturer's instruction. RNA quantities were assessed on a Nanodrop 1000 (Thermo Fisher). RNA quality was checked on RNA Nano chips on the Agilent 2100 Bioanalyzer (Agilent Technologies). All samples resulted in RIN values over 7 and intact 18S and 28S bands.

For mRNA quantification, RNA was transcribed to cDNA using the Qiagen QuantiTect Kit according to the manufacturer's instructions and qPCR was performed using the Qiagen qPCR Sybr Green Kit with gene specific primers (connexin 43 forward: GACAGGTCTGAGTGCCTGAA, reverse: TGCCCGGACACTACTCTTTC, CXCL12 forward: GCCAACATCAAGCATCTCAA, reverse: AGAGAGTGGGACTGGGTTTG, beta-Actin forward TCAACACCCAGCCATGTAC, reverse: CTCCGGAGTCCATCACGATG) on an Applied Biosystems 7500 Fast qPCR system (Thermo Fisher). The mean of two technical replicates was used for each individual sample. A standard curve was prepared and run for each gene for assessing PCR efficiency. Expression quantities are expressed relative to beta-Actin.

Sequences of pig miRNAs were derived from the miRBase database. For miRNA quantification small RNAs were transcribed to cDNA using the Qiagen miScript II RT Kit according to the manufacturer's instructions and qPCR was performed using the Qiagen qPCR Sybr Green Kit with the Universal Primer and an miRNA specific primer (mir-1: GCAGTCTGGAATGTAAAGAAG, mir-29a:, let7a: GCAGTGAGGTAGTAGGTTGT, miR-126: CGACGTCGTACCGTGAGTAA, miR-146a: CGTAGGTGAGAACTGAATTCC). The mean of two technical replicates was used for each individual sample. Expression rates were corrected for PCR efficiencies using dilution curves and analyzed in relation to let7a expression.
